# Population Pharmacokinetics and Dose Optimization of Ceftazidime and Imipenem in Patients with Acute Exacerbations of Chronic Obstructive Pulmonary Disease

**DOI:** 10.3390/pharmaceutics13040456

**Published:** 2021-03-27

**Authors:** Thu-Minh Nguyen, Thu-Hue Ngo, Anh-Quan Truong, Dinh-Hoa Vu, Dinh-Chi Le, Ngan-Binh Vu, Tuyet-Nga Can, Hoang-Anh Nguyen, Thu-Phuong Phan, Françoise Van Bambeke, Céline Vidaillac, Quy-Chau Ngo

**Affiliations:** 1 Department of Pharmacy, Bach Mai Hospital, Hanoi 11519, Vietnam; nguyenminh802002@yahoo.com (T.-M.N.); ngaduoc@gmail.com (T.-N.C.); anhnh@hup.edu.vn (H.-A.N.); 2 National Drug Information and Adverse Drug Reaction Monitoring Centre, Hanoi University of Pharmacy, Hanoi 11021, Vietnam; 1401263@hup.edu.vn (T.-H.N.); 1401502@hup.edu.vn (A.-Q.T.); 3 Department of Analytical Chemistry, Hanoi University of Pharmacy, Hanoi 11021, Vietnam; child@hup.edu.vn (D.-C.L.); binhvn@hup.edu.vn (N.-B.V.); 4 Respiratory Center, Bach Mai Hospital, Hanoi 11519, Vietnam; thuphuongdr@gmail.com (T.-P.P.); ngoquychaubmh@gmail.com (Q.-C.N.); 5 Louvain Drug Research Institute, Université catholique de Louvain, B1.73.05, B-1200 Brussels, Belgium; francoise.vanbambeke@uclouvain.be; 6 Center for Tropical Medicine and Global Health, University of Oxford, Oxford OX3 7LG, UK; cvidaillac@oucru.org; 7 Oxford University Clinical Research Unit, University of Oxford, Ho Chi Minh City 72700, Vietnam

**Keywords:** chronic obstructive pulmonary disease, acute exacerbations, ceftazidime, imipenem, dose optimization, *Pseudomonas aeruginosa*

## Abstract

Background: Ceftazidime and imipenem have been increasingly used to treat Acute Exacerbations of Chronic Obstructive Pulmonary Disease (AECOPD) due to their extended-spectrum covering *Pseudomonas aeruginosa*. This study aims to describe the population pharmacokinetic (PK) and pharmacodynamic (PD) target attainment for ceftazidime and imipenem in patients with AECOPD. Methods: We conducted a prospective PK study at Bach Mai Hospital (Viet Nam). A total of 50 (ceftazidime) and 44 (imipenem) patients with AECOPD were enrolled. Population PK analysis was performed using Monolix 2019R1 and Monte Carlo simulations were conducted to determine the optimal dose regimen with respect to the attainment of 60% and 40% *f*T>MIC for ceftazidime and imipenem, respectively. A dosing algorithm was developed to identify optimal treatment doses. Results: Ceftazidime and imipenem PK was best described by a one-compartment population model with a volume of distribution and clearance of 23.7 L and 8.74 L/h for ceftazidime and 15.1 L and 7.88 L/h for imipenem, respectively. Cockcroft–Gault creatinine clearance represented a significant covariate affecting the clearance of both drugs. Increased doses with prolonged infusion were found to cover pathogens with reduced susceptibility. Conclusions: This study describes a novel and versatile three-level dosing algorithm based on patients’ renal function and characteristic of the infective pathogen to explore ceftazidime and imipenem optimal regimen for AECOPD.

## 1. Introduction

Chronic obstructive pulmonary disease (COPD) is a condition where progressive and irreversible structural changes of the airways lead to airflow obstruction and persistent respiratory symptoms including shortness of breath, cough, and expectorations [1]. COPD is mainly caused by exposure to harmful particles or fumes such as tobacco smoking and air pollution, two major and global burdens [1]. With an estimated 328 million people presenting moderate to severe COPD worldwide, the condition is considered as a major health and economic concern globally, and more specifically in low- and middle-income countries (LMICs) where almost 90% of worldwide COPD deaths are reported [2,3]. Approximately 7.1% of the Vietnamese population (nearly 6.8 million people) have COPD [4]. These patients occupy roughly 25% of the beds in the respiratory wards of hospitals, at our institution [5], and nationwide [4].

Due to the heterogeneity of its pathogenesis and its overlapping clinical manifestations, COPD is difficult to manage [6]. Patients are at risk of experiencing sudden episodes of breathing difficulty associated with a chesty cough, significantly affecting the progression of the disease and the patients’ quality of life [7]. Patients with acute exacerbations of COPD (AECOPD) are at higher risk of treatment failure [8], future readmissions [9], and death [10]. Unfortunately, these events are difficult to predict and control due to their large variety of etiologies and clinical presentations [11,12]. Up to 30% of the AECOPD are idiopathic, while 50 to 70% are triggered by respiratory viral infections [13,14,15,16]. Concomitant short-term antibiotic therapy is however indicated when patients with AECOPD present an increase in dyspnea and production of purulent sputum, a sign of a growing bacterial burden in the airways [1]. The choice of antibiotic is based on the local bacterial resistance epidemiology [1,12] with typically an initial empirical treatment with aminopenicillin combined with clavulanic acid, or a macrolide or tetracycline agent [1]. For patients with a history of frequent exacerbations, severe airflow obstruction, and/or requiring mechanical ventilation, microbiological analysis is recommended to identify the pathogen present and its drug susceptibility profile [1,12].

*Pseudomonas aeruginosa* is one of the most common species found in the sputum of patients with AECOPD [17,18]. It is unclear whether it is a cause or a consequence of AECOPD, however, it is recognized that its presence in the sputum of patients represents a risk factor for severe exacerbation, poorer clinical outcomes, prolonged hospitalization, and increased cost [19,20]. Despite the lack of clinical evidence to demonstrate the benefit of targeting *P. aeruginosa* to reduce the risk of exacerbations, anti-*Pseudomonas* agents are commonly used to treat severe AECOPD where the pathogen is suspected or confirmed [21]. At our institution where *P. aeruginosa* is commonly found in our patients’ population [22], anti-*Pseudomonas* drugs including ceftazidime (CAZ), imipenem (IMI), meropenem, piperacillin/tazobactam are used to manage over 50% of hospitalized COPD patients, whether microbiological results are available or not [23]. Epidemiology studies performed at our institution have revealed that local strains of *P. aeruginosa* are mainly susceptible to CAZ and IMI. This observation has guided our physicians to use these 2 antimicrobials routinely but has also raised the concern of their optimal usage in the AECOPD patients to preserve their efficacy and reduce the pressure of selection [22,23].

Clinical/pharmacokinetic prediction models are powerful tools to guide infectious disease physicians and pharmacists in the selection of optimal dosing regimens [24]. Limited clinical data are available on the pharmacokinetics (PK) and pharmacodynamics (PD) of CAZ and IMI in patients presenting AECOPD. It is therefore difficult to confirm that the doses currently used in this population are optimal. The aims of this study are to 1) collect CAZ and IMI PK data in AECOPD patients, 2) develop a PK model that best describes the time course of drug exposure in this specific population, and 3) describe how patients and pathogen characteristics influence optimal dosing regimens of these 2 antibiotics. The long-term goal of this study is to raise our physicians but also the global community’s awareness of the existing gap(s) in terms of CAZ and IMI use and dose optimization to better manage AECOPD and reduce the risk of emergence of antimicrobial resistance.

## 2. Materials and Methods

### 2.1. Study Design and Participants

This study was prospectively performed at the Respiratory department (116 beds) of Bach Mai Hospital (2400 beds) between August 2018 and March 2019. To be eligible, patients must be (1) aged over 18, (2) presenting AECOPD matching the definition of the Global initiative for chronic obstructive lung disease 2020 (i.e., an acute worsening of respiratory symptoms that results in additional therapy) [1] and (3) receiving CAZ or IMI therapy for at least three consecutive days. Only patients who did not sign a consent were excluded. The study was conducted according to the guidelines of the Declaration of Helsinki and approved by the Institutional Review Board of Bach Mai hospital (reference # 2919/QD-BM for the protocol BM-2017-957-50, approval date on 26 December 2017). Written informed consent was obtained from all patients or their legal representatives.

### 2.2. Sample Collection and Analysis

Prescription and dosing regimens (IMI: 0.5 g q12h, 0.5 g q8h, 0.5 g q6h, 1 q q12h and 1 g q8h; CAZ: 1 g q12h, 1 g q8h, 1 g q6h, 2 g q12h and 2 g q8h) were at the discretion of the physicians based on routine care practices. Clinical data including patient characteristics, antibiotic used, and microbiological results were collected at baseline and on the days of PK sampling. Due to cultural challenges, our pharmacokinetic study used a sparse sampling strategy with 2 samples per patient. Samples were collected at least 30 min after infusion of the third dose to ensure a steady-state was attained and one to two hours prior to the 4th dose. Blood samples (3 mL) were collected into a heparinized vacutainer and centrifuged immediately to obtain plasma. To improve stability of IMI, plasma sample (1 mL) was mixed (1:1, *v/v*) with 0.5 M 3-morpholino-propane-sulphonic acid buffer (MOPS, pH 6.8, Sigma-Aldrich, Co., St. Louis, MO, USA) and stored at −40 °C until sample analysis. Analysis was performed within 1 week at the Department of Analytical Chemistry and Toxicology of Hanoi University of Pharmacy to determine CAZ and IMI concentrations by validated high-performance liquid chromatography using Agilent 1200 equipped with a PDA detector (Agilent Technologies, Santa Clara, CA, USA) [25,26].

For quantification of CAZ concentration, 100 µL aqueous solution of cefepime (National Institute of Drug Quality Control, Hanoi, Vietnam) (200 µg/mL) as internal standard was mixed to 200 µL of plasma. Acetonitrile (Merck KGaA, Darmstadt, Germany) (500 µL) was added to the mixture for protein precipitation. The sample was then vortexed for 30 s and centrifuged at 14,000 rpm for 5 min. The resulting supernatant (800 µL) was transferred into an Eppendorf tube containing 500 µL of chloroform. The tube was vortexed for 30 s and then centrifuged at 1700 rpm for 5 min. A sample (20 µL) of the upper aqueous extract was injected into the Agilent 1200 chromatography system using an Inerstil^®^ ODS −3 column (GL Sciences Inc., Tokyo, Japan) (250 mm × 4.6 mm; 5 μm). The mobile phase consisted of a mixture (9:1, *v*:*v*) of sodium dihydrogenophosphate (Merck KGaA, Darmstadt, Germany) (50 mM, pH 3.2) and acetonitrile that was maintained at a flow rate of 1.5 mL/min. UV detection was set at 260 nm for recording chromatograms. The method was proven to be reproducible with bias of 1.8%, 7.2%, −6.1% and 1.4% and precision of 5.5%; 2.1%, 2.5%, and 2.2% at concentrations of 2, 6, 50, and 80 µg/mL, respectively. The lower limit of quantification was 2 µg/mL and the method was linear over the range of 2 to 100 µg/mL [25].

For quantification of IMI concentrations, 100 µL of meropenem (20 µg/mL) as internal standard was mixed to 200 µL of plasma and 200 µL of 0.5 M MOPS buffer pH 6.8. Acetonitrile (500 µL) was added to the mixture for protein precipitation. The sample was then vortexed for 30 s and centrifuged at 6500 rpm for 10 min. The resulting supernatant (500 µL) was evaporated under a nitrogen stream and the residual was dissolved in 200 µL 0.5 M MOPS buffer pH 6.8. A sample (50 µL) of the resulting solution was injected into a Supelco Ascentis^®^ C8 guard column (Supelco, Bellefonte, PA, USA) (20 × 4 mm; 5 µm) and a C8 Supelco Ascentis^®^ C8 HPLC column (150 × 4.6 mm; 5µm) (Supelco, Bellefonte, PA, USA). The analytes were eluted at a flow rate of 1 mL/min with ultraviolet detection at 298 nm using a mobile phase consisting of phosphate buffer (0.05 M, pH 7.4) and methanol. The proportion of phosphate buffer in solvent gradient was as followed: 0–4 min: 96%; 4–7 min: decrease from 96% to 30%; 7–9 min: stable at 30%; after 9 min: increase from 30% to 96%. The method was proven to be accurate and precise with bias of 0.9%, −11.8%, −2.5%, and 5.45% and precision of 7.5%, 3.2%, 9.9%, and 10.7% at concentrations of 0.5, 1, 20, and 40 µg/mL, respectively. The lower limit of quantification was 0.5 µg/mL and the method was linear over the range of 0.5 to 50 µg/mL [26].

### 2.3. Population Pharmacokinetic Modelling

Population PK analysis was performed using MONOLIX software (Monolix version 2019R1. Antony, France: Lixoft SAS, 2019.). Population PK parameters were estimated by maximum likelihood using Stochastic Approximation Expectation-Maximization (SAEM) algorithm [27].

The basic population PK model included a combination of structural and statistical models. The structural PK models consisted of one- and two-compartment systems with first-order elimination, whereas the statistical PK models consisted of systems where individual PK parameters were assumed to follow log-normal distributions [27] and where exponential random effects were applied for inter-individual variabilities as followed:

Pi = P × e^ηPi^ (1)

Pi and η^Pi^ represent the PK parameters of subject i and its individual random effect, respectively. The distribution of Pi was defined by two components P and **ω**P, which were the typical value of the parameters and the standard deviation of η^Pi^, respectively. Additionally, independent random effects corresponding to a diagonal of the variance-covariance matrix were assumed. The constant, proportional, and combined error models were assessed.

The appropriate basic model was selected based on the Bayesian Information Criterion (BIC) [28], the precision of estimates, and the goodness-of-fit plots. The BIC was calculated as follow BIC = −2log(L) + k(logN), in which L was a likelihood, k was the total number of parameters (i.e., fixed effects, random effects, and error model parameters) in the model, and N was the total number of data observations. The model with the lowest BIC was selected [28].

Covariates tested included age, weight, body mass index (BMI), gender, clearance creatinine estimated by Cockcroft and Gault (CLCR_CG_) [29] and MDRD-4 equations [29], Anthonisen score, respiratory distress, and diuretics intake. The covariates were measured at the time of blood sampling, except for the Anthonisen score, which was recorded on the first day the patient entered the Respiratory Centre. Continuous covariates were log-transformed and centered as followed:

logtCOVi = log(COVi/COVweighted mean)(2)

where COV weighted mean is the mean of the covariate weighted by a number of observations per individual. The covariate and PK parameter relationships were visually investigated in MONOLIX. The selection of covariates was determined using a stepwise approach as described previously [28]. First, the correlation between the covariates and the PK parameters were preliminary evaluated using a visual graph and univariate statistics. The covariates with a *p*-value less than 0.05 were considered for the covariate model. Second, in forward-selection, covariates were added to the model. The covariates with an objective function value (OFV) reduction greater than 6.635 were considered to be significant (*p* < 0.01). Third, in backward elimination, any covariate associated with an OFV increase greater than 10.828 (*p* < 0.001) was kept in the model [28]. Model adequacy was further evaluated using goodness-of-fit. Observations values were plotted versus individual and population prediction values. The individual weighted residuals (IWRES) and population-weighted residuals (PWRES) were plotted versus predicted concentration plots, and the normalized prediction distribution errors (NPDE) versus time after the dose to evaluate for randomness around the line of unity [28]. The uncertainty of the population parameters of the final model was finally estimated using 1000 bootstrap replicates. The predictive performance of the developed model was examined using a visual predictive check (VPC) plot [28].

### 2.4. Monte Carlo Simulations and Development of a Therapeutic Algorithm

Monte Carlo simulations of 1000 patients were applied with different dosing regimens for CAZ and IMI using the final model. CAZ dose of 1 g q12h, 1 g q8h, 2 g q12h, and 2 g q8h and IMI dose of 0.5 g q6h, 0.75 g q6h, 1 g q6h, and 1 g q8h were used in the simulations [30,31]. A short-term infusion (SI) of 30 min and extended infusion (EI) of 3 h were applied. Continuous infusion (CI) of CAZ and IMI were also examined. CI of CAZ consisted of a loading dose of 2 g followed by a CI of 6 g q24h. CI of IMI consisted of a loading dose of 1 g followed by a CI of 4 g q24h, renewed every 3 h due to the rapid degradation of the molecule in solution [30,31]. The estimated glomerular filtration rates of 30–60, 60–90, and >90 mL/min were used to stratify the simulations. The fraction of time that free drug concentration remains above Minimum Inhibitory Concentration (*f*T > MIC) was used as the surrogate PK/PD index for both CAZ and IMI. Free concentrations of the drug were assumed using the protein binding values of 14% for CAZ [32] and 20% for IMI [33]. The probability of target attainment (PTA) aiming at 40% *f*T>MIC for IMI and 60% *f*T>MIC [34,35,36] for CAZ, was estimated at 72 hrs using MIC ranging from 0.125 to 32 µg/mL. These PTA were selected as they have previously been associated to significantly reduce treatment failure [37,38]. A more aggressive target of 100% *f*T>MIC was also examined to cover scenarios where patients present severe conditions [39,40]. *P. aeruginosa* MIC breakpoints considered for susceptibility (S), intermediate susceptibility (I), and resistance (R) followed the guidance of Clinical & Laboratory Standards Institute (CLSI) and were ≤8 mg/L (S), 16 mg/L (I) and ≥32 mg/L (R) for CAZ and ≤2 mg/L (S), 4 mg/L (I) and ≥8 mg/L (R) for IMI [41]. Sufficient antimicrobial effect was assumed if PTA exceeded 90% (90% PTA) [42]. The lowest daily dose obtaining ≥ 90% PTA was considered to be optimal [42]. Three clinical microbiology scenarios were then assumed for dose selection including (1) *P. aeruginosa* confirmation without susceptibility result or high risk of *P. aeruginosa* infection; (2) *P. aeruginosa* infection with antibiogram result (susceptible, intermediate, and resistant) and (3) *P. aeruginosa* infection with MIC values known. All simulations were performed using package mlxR (Simulx, RRID:SCR 000486) version 4.1.0 [43] in R 4.0.3 (R Core Team, 2020).

## 3. Results

### 3.1. Demographics

Key patient demographic and baseline characteristics are summarized in Table 1. A total of 94 patients, 50 receiving CAZ and 44 receiving IMI, were included in the study. Patients between the 2 groups did not present significant demographic differences, except for CLCR_CG_. Median (interquartile range, IQR) age was 69 yo (63–77) and 65 yo (60–72) for patients in the CAZ and IMI groups, respectively. Median (IQR) of total body weight and free fat masses were 51 kg (47–57) and 45 kg (41–47) for patients in the CAZ group, and 50 kg (47–55) and 43 kg (40–46) for patients in the IMI group, respectively. The median (IQR) of CLCR_CG_ was 62.9 mL/min (49.0–76.8) for CAZ patients versus 76.6 mL/min (57.5–96.6) for IMI patients. In both groups, over 60% of the patients had a history of COPD, and few (2 and 9% for CAZ and IMI, respectively) had COPD for more than 10 years. Forced expiratory volume (FEV1) values at baseline were available for 23 and 6 patients in CAZ and IMI groups, respectively, and showed that patients had overall poor pulmonary function (>90% had FEV1 < 70%). A significant number of the patients also presented respiratory distress (34 and 66% in CAZ and IMI groups, respectively) and had invasive ventilation (14 and 30% in CAZ and IMI groups, respectively). However, no patient required admission to the Intensive Care Unit during the study period. All patients received inhaled and/or systemic bronchodilators. The median antibiotic treatment duration for both drugs was 10 days with a value ranging from 8–13 or 7–14 in the CAZ or IMI group, respectively. Conventional dosing interval and infusion time followed the decision of the physician and the sampling varied accordingly. Dose of 1 g every 8 h was used in 38 (78%) patients with CAZ and 24 (55%) patients with IMI.

### 3.2. Model Building Process

A total of 97 and 84 plasma samples were obtained from patients using CAZ and IMI, respectively. One sample only was obtained in seven patients (five patients refused the second blood sampling, one patient transferred out after the first blood sample, and one sample could not be collected due to emergency care). The infusion times were different between patients. The concentrations of CAZ and IMI recovered in patient blood samples are presented in Supplemental Figure S1. The result of basic PK model development is presented in Supplemental Table S1. For both drugs, the smallest BIC values (613.47 and 552.06 for CAZ and IMI, respectively) were observed in a one compartmental model with first-order elimination, proportional error, and log-normal parameter distribution. The covariate models derived from the stepwise procedure are presented in Supplemental Table S2. No covariate showed a significant impact on the Vd of both drugs. In contrast, the CLCR_CG_ was a significant covariate on the CL of both CAZ and IMI, with a reduction of OFV greatest in comparison to other significant covariates. Adding other covariates did not further significantly improve the OFV.

### 3.3. Population Pharmacokinetic Model

**Figure 1:**
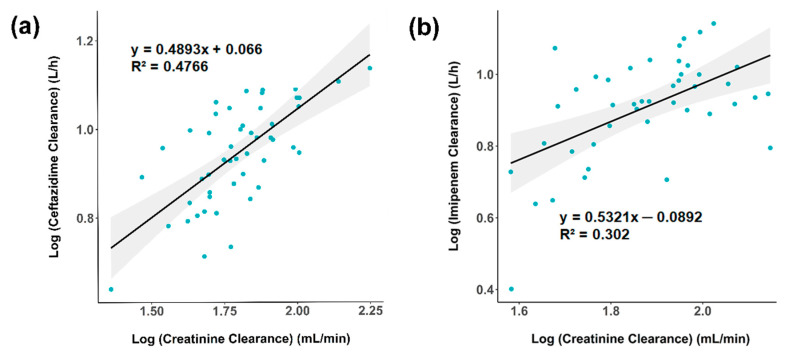
Individual ceftazidime. (**a**) and imipenem (**b**) clearance estimates versus creatinine clearance estimated according to Cockcroft and Gault. Log transformed values are plotted, with the solid lines and grey shading areas illustrating the linear regression and the 95% confident interval, respectively. The regression equations and respective correlation coefficients (R^2^) are presented for each plot. Creatinine clearance were estimated by Cockcroft and Gault equation (CLCR_CG_).

Table 2 summarizes the population PK estimates and bootstrap results for CAZ and IMI in the selected model. The population estimates of the Vd and CL were 23.7 L and 8.74 L/h for CAZ, and 15.1 L and 7.88 L/h for IMI, respectively. For random effect, the inter-individual variability (IIV) of Vd and CL were 13% and 20.8% for CAZ, and 12.9% and 30% for IMI, respectively. The correlation between CLCR_CG_ and CL of CAZ and IMI is illustrated in Figure 1. The effect of CLCR_CG_ on CL of CAZ and IMI was as followed: CAZ: CLi = 8.74 × (CLCRi/69.02)^0.485^ × e^ηCL^; IMI: CLi = 7.88 × (CLCRi/75.54)^0.532^ × e^ηCL^. Bootstrapping results of the 1000 replicates showed marginal differences from respective estimates in the final model (Table 2).

### 3.4. Model Evaluation

**Figure 2:**
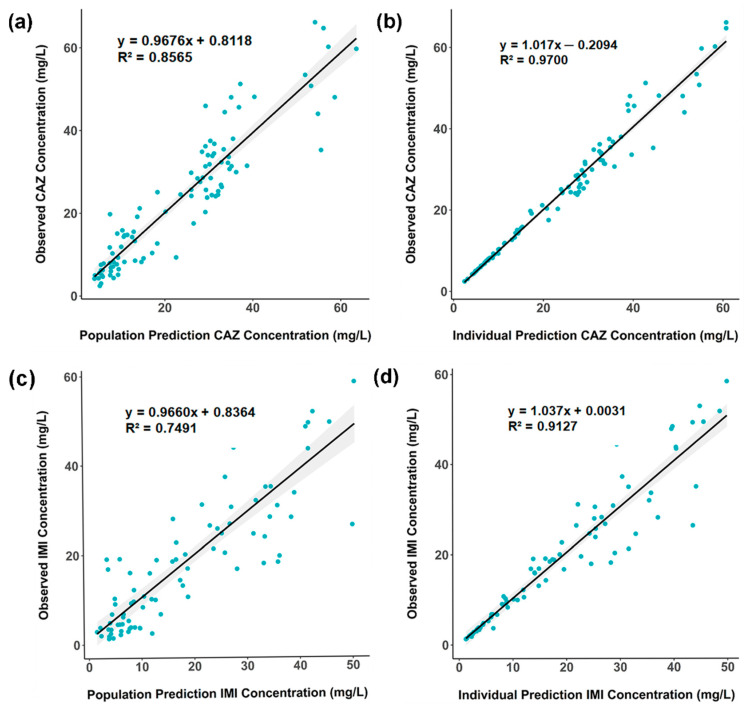
Basic goodness-of-fit plots from the final covariate model. (**a**,**b**) Measured ceftazidime (CAZ) concentrations are plotted against the population (**a**) or the individually-fitted (**b**) concentrations. (**c**,**d**) Measured imipenem (IMI) concentrations are plotted against the population (**c**) or the individually-fitted (**d**) concentrations. The solid lines represent linear regression lines with shade areas illustrating the 95% confident intervals. The regression equations and respective correlation coefficients (R^2^) are presented.

**Figure 3:**
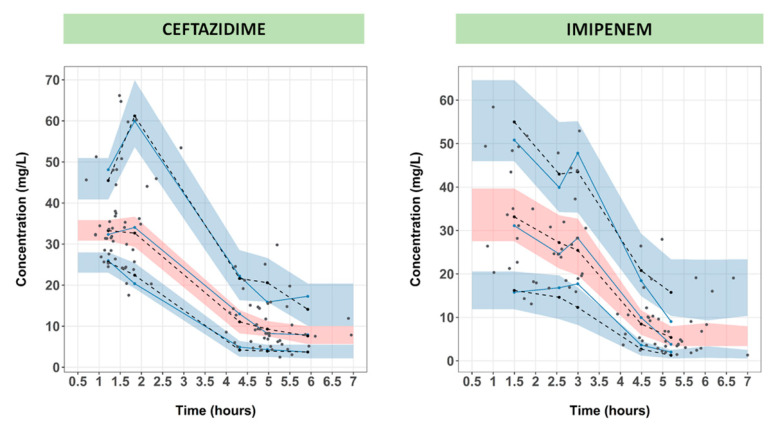
Visual Predictive Check plot versus time. The grey solid lines indicate the 10th, 50th, and 90th percentiles of the observed data. The grey-black dashed lines indicate the 10th, 50th, and 90th percentiles of simulated data. The shaded grey and pink areas represent 90% prediction intervals from the corresponding percentiles as predicted by the model.

The basic goodness-of-fit plots representing the correlation between observed vs. individual or population predicted concentrations are displayed in Figure 2. Population predicted concentrations showed a good correlation with observed concentrations (correlation coefficients of 0.86 and 0.75 for CAZ and IMI, respectively). Population weighted residuals (PWRES), individual weighted residuals (IWRES), and Normalized Prediction Distribution Error (NDPE) plots showed no significant bias for both drugs as the data equally distributed around the horizontal axis (Supplemental Figure S2). Visual Predictive Check (VPC) plots suggested that the median and 5th and 95th percentiles of observed concentrations were properly predicted by the respective bootstrapped 95% confident intervals (Figure 3).

### 3.5. Monte Carlo Simulations and Dosing Regimen Recommendations

**Figure 4:**
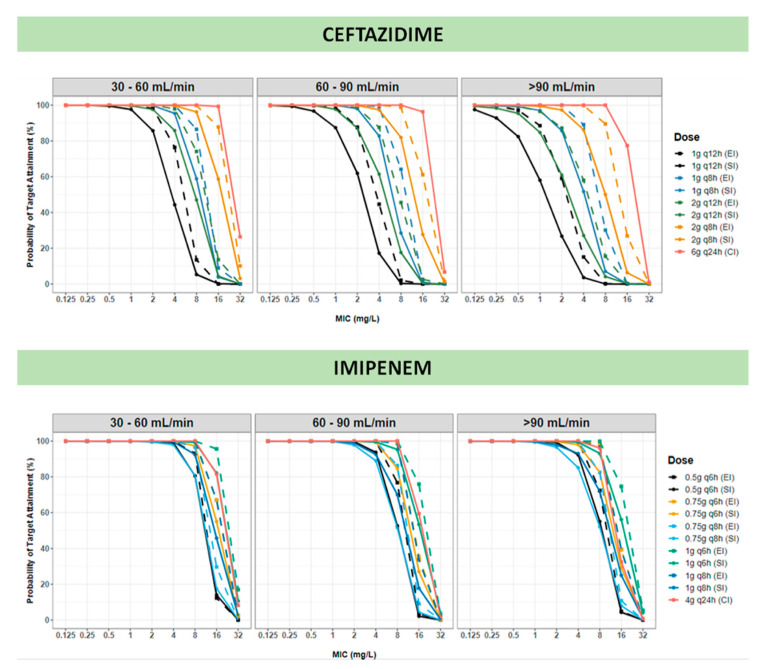
Probability of target attainment (PTA) of ceftazidime with a target of 60% *f*T>MIC and imipenem with 40% *f*T>MIC. MIC values based on the recommendation of Clinical and Laboratory Standards Institute 2020 (CLSI 2020); Dose regimens using short-term infusion (SI: 0.5 h), extended infusion (EI: 3 h), and continuous infusion (CI) are simulated. The simulations were stratified based on CLCR_CG_.

Figure 4, Supplemental Tables S3 and S4 illustrate PTA values for various CAZ and IMI dose regimens using targets of 60–100% *f*T>MIC for CAZ and 40–100% *f*T>MIC for IMI. Simulations were performed and analyzed based on patient renal function. For both drugs, no practical dose covered highly resistant pathogens with MIC equal or greater than 32 mg/L. Patients with good renal function (ie CLCR_CG_ > 90 mL/min) might not reach the threshold of 90% PTA on pathogens exhibiting high MIC values, especially when low dose and short infusion were applied. For CAZ, CI showed improved PTA compared to EI. For both drugs, SI (30 min) appeared inferior to EI and CI, so it was not further considered.

**Figure 5:**
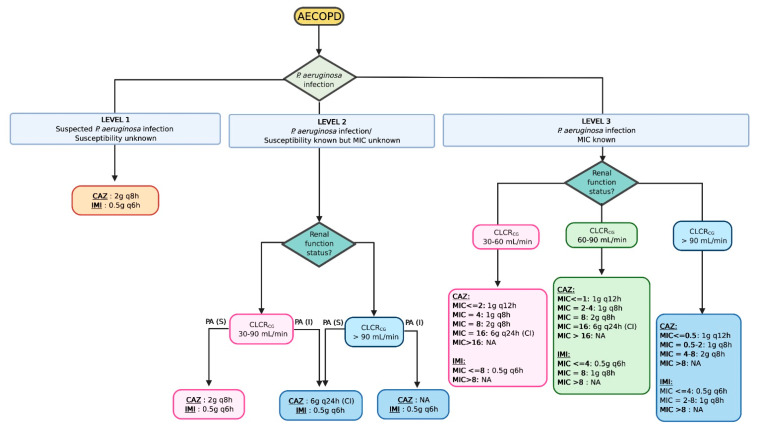
Proposed dosing algorithm based on the simulation results of ceftazidime and imipenem in Acute Exacerbation of Chronic Obstructive Pulmonary Disease (AECOPD) to achieve 60% *f*T>MIC for ceftazidime and 40% *f*T>MIC for imipenem. MIC: Minimum inhibitory concentration, PA: *Pseudomonas aeruginosa*, (S): Susceptible; (I): Intermediate resistance, IMI, imipenem; CFZ, ceftazidime; CLCR_CG_, Clearance creatinine according to Cockcroft and Gault equation; q6h, q8h, and q12h, dose following 3 h extended infusion with dose interval of 6, 8, or 12 h, respectively; CI, continuous infusion. Created with Biorender.com (accessed on 1 February 2021).

Based on the results of our simulations and the selection of our model, three microbiological scenarios were assumed to find the most appropriate dosing strategy when *P. aeruginosa* infection is suspected and targeted. Figure 5 and Supplemental Figure S3 illustrate 2 dosing algorithms depicted from our simulations based on the susceptibility profile of the pathogen. For CAZ, regimens using CI were required to cover pathogens with intermediate resistance. When targeting 100% *f*T>MIC, prolonged infusion (either EI or CI) increased the chance to obtain 90% of PTA for both CAZ and IMI. (Supplemental Tables S3 and S4 and Figure S3). Daily doses of 6 g CAZ or 4 g IMI as CI were required to cover most of the susceptible strains. For intermediate resistant and even pathogens with MIC of 8 mg/L, IMI daily dose of 4 g as CI was likely sufficient. No clinical dose of CAZ, even in CI, could cover pathogens with intermediate resistance (Supplemental Tables S3 and S4 and Figure S3).

## 4. Discussion

The use of antimicrobial agents to treat AECOPD remains controversial and challenging [1,6,11,12,13,14,15,16]. Since acute exacerbations are triggered by viral multiple factors including viral, bacterial infections, or non-infectious causes, the benefit of antimicrobial treatment remains unclear and subject of debate [44]. However, when the presence of *P. aeruginosa* is suspected or confirmed in the patient sputum or the patient presents severe dyspnea, guidelines recommend the use of antimicrobials, the selection of which varies depending on local epidemiology [1,6]. A recent meta-analysis suggested that using antibiotics, especially targeting *P. aeruginosa*, reduces the treatment failure and shortens the hospital length of stay of patients with AECOPD [41,45]. In Vietnam, CAZ and IMI are routinely used as they have proven to retain efficacy against the local strains of *P. aeruginosa* [23]. However, concerns are rising towards their proper use to preserve their efficacy and prevent the emergence of resistance. Pathophysiological and clinical factors related to COPD might affect PK profiles of antimicrobials; hence an inappropriate dosing regimen could result in treatment failure, increased emergence of resistance, and higher mortality [19]. Designing an adapted dosing regimen, optimized for specific populations, especially critically-ill patients such as those presenting AECOPD, remains crucial. To the best of our knowledge, this study is the first one to model and simulate CAZ and IMI PKs to explore optimal dosage regimens in patients presenting AECOPD.

In the context of our study, which included 50 and 44 patients in CAZ and IMI groups, respectively, a one-compartment model with first-order elimination, proportional error model with CLCR_CG_ as a covariate on clearance provided a superior fit to the data. This result is consistent with previous works published in cystic fibrosis (CF) and critically-ill patients [46,47]. In this study, the Vd of CAZ was 23.7 L. This is comparable with that observed in patients with nosocomial pneumonia (Vd of 23.1 L) [48], and only marginally larger than what is reported in the healthy population (15–20 L) [49]. Similarly, CAZ CL was 8.74 L/h in this study versus 6.47 L/h in patients with nosocomial pneumonia [48] or 8.57 L/h in CF patients [49]. These values are also in line with the CL reported in healthy volunteers (8.77 L/h) [46], suggesting that the renal function rather than other covariates might predict the elimination of CAZ observations were confirmed in the covariate model where CLCR_CG_ was the only covariate showing a linear relationship with CL of CAZ when log transforming both sides of the equation (correlation coefficient 0.4766). This finding was also observed in critically-ill patients [48,50]. For IMI, the estimated Vd was 15.1 L in this study, a value comparable with that observed in a pooled population (15.8 L) [33], whereas larger Vd were reported in ventilator-associated pneumonia patients (20.4 L) [51], or sepsis patients with a high burden of IV fluid (Vd 29.9 L) [52]. IMI CL in this study was 7.88 L/h versus 13.2 L/h in VAP patients [51]. However, similar to CAZ, CLCR_CG_ also had a significant impact on IMI CL (correlation coefficient of 0.302).

Consistent with the literature, our results confirm that CAZ and IMI elimination is significantly impacted by patients’ renal function (OFV reduction of 26.26 and 11.89 for CAZ and IMI, respectively) [33,48,50,51]. To acknowledge this observation, we further explored dose optimization and designed dosing recommendations stratified according to CLCR_CG_. It should be noted that most COPD patients were elderly with chronic conditions, and therefore the chance of augmented renal clearance was low [53]. Therefore, the use of high doses as recommended in other critically ill patients with sepsis should be taken cautiously [54]. We performed simulations with conventional daily doses of 2 to 6 g for CAZ and 2 to 4 g for IMI. Higher doses of CAZ (2 g q8h and 3h EI) were also applied to increase the likelihood to cover *P. aeruginosa* strains with reduced susceptibility [55]. According to CLSI, CAZ intermediate resistant *P. aeruginosa* exhibits a MIC value of 16 mg/L. Based on our study, no practical dose could reach the target of 60–100% *f*T>MIC for patients with normal renal function when intermediate resistance was involved. However, a daily dose of 6 g administered in CI could reach the target >60% *f*T>MIC for patients with impaired renal function and intermediate resistant isolates. In any case, no simulation was able to cover resistant strains with MIC greater than 16 mg/L, and different treatment strategy including other antibiotics should be considered in such cases [56]. In contrast, for IMI the target of 40% *f*T>MIC could be achieved with a dose as low as 0.5 g q6h EI for susceptible pathogens, and 4 g q24h for a pathogen with a MIC equal or greater than 8 mg/L when the patient had normal renal function. Therefore, the dose of 1 g q8h previously suggested to cover MDR *P. aeruginosa* might not be appropriated for all situation*s* [55]. A more aggressive target of 100% *f*T>MIC was also investigated as it is sometimes suggested for critically-ill patients [39]. In this study, we show that to achieve such PTA, a dose of 4 g q24h administered as CI is required (Supplemental Figure S4). Unlike intermittent infusion, CI in critically ill patients allows for antibiotic concentration to remain plateau [57] hence making it easier to attain the target of 100% *f*T>MIC and cover susceptible pathogens. However, in the case of pathogens with reduced susceptibility, when using CI and a low dose of antibiotic, the concentration might never reach the MIC level, increasing, therefore, the risk of treatment failure and emergence of resistance [31]. To address that, our algorithm uses only CI with a high dose of antibiotics. This represents a challenge in practice since IMI is poorly stable in aqueous fluid requiring therefore regular renewal of the infusion fluid [58]. IMI CI might be considered when patients present severe clinical conditions or resistant pathogens are suspected and no other treatment options are available [31].

The use of CAZ and IMI is common in medical settings like our hospital [22,23]. As observed in this study, the dose, dose interval, and infusion time vary greatly between patients with no clear recommendation to guide our physicians. There is therefore a possibility of under-dosing patients, increasing the risk of treatment failure, prolonged hospital stay, mortality, and promoting the emergence of resistance, an issue that our hospital is already facing [59]. To better understand the role played by antibiotics in the management of AECOPD, further research including PK/PD modeling and Monte Carlo simulations are much warranted [60]. This is the first study to provide insights towards a better understanding of the impact of dose and dose interval on the predicted efficacy of IMI and CAZ in patients with AECOPD. This study carries, however, several limitations, that may to a certain extent impact the results. First, due to cultural challenges related to blood sampling for research purposes, the PK study was performed with a sparse sampling strategy. This may affect the precision of the developed population pharmacokinetic models [61]. However, our PK models were relatively comparable with other published ones, suggesting the appropriateness for dose simulation. Second, patients included in our study present smaller sizes and lower weights compared to the Caucasian population. This should be taken into consideration for future extrapolation of the data to caucasian patients and future PK/PD studies should be required to confirm our finding. Third, CLCR_CG_ was calculated to estimate the patient’s renal function, instead of using timed urine collection. We believe that the bias it may have induced in our study is only marginal as our population did not include critically severe patients. However, further studies in critically ill patients should consider the benefit of using timed urine collection. Last, due to the lack of sufficient MIC data of pathogens isolated from COPD patients in our hospital, we were not able to translate and compare our findings with the current local practices. We have therefore used the CLSI classification to examine the chance of attaining the PK/PD target. The empirical doses in the proposed algorithm derived from our simulations were applied for pathogens with MIC below the susceptible breakpoint, and the chance to cover pathogens in actual patients may be different.

## 5. Conclusions

Our study described PK characteristics of CAZ and IMI, two of the most commonly used anti-*Pseudomonas* beta-lactams, in hospitalized COPD patients presenting acute exacerbation. We propose a dosing algorithm in which we highlight the use of high-dose CAZ for suspected *P. aeruginosa* with reduced susceptibility. Additional studies are warranted to validate our proposed dosing algorithm and to make it applicable in clinical practice.

## Figures and Tables

**Table 1:** Personal and clinical characteristics of patients with acute exacerbations of chronic obstructive pulmonary disease enrolled in the ceftazidime and imipenem cohorts.

Parameters	Ceftazidime (*n* = 50)	Imipenem (*n* = 44)
**Age (years)**	69 (63–77)	65 (60–72)
**Male (No., %)**	47 (94)	41 (93)
**History of diagnosis of COPD**		
<1 year (No., %)	18 (36)	10 (23)
1–10 year (No., %)	31 (62)	30 (68)
>10 year (No., %)	1 (2)	4 (9)
**Total body weight (kg)**	51 (47–57)	50 (47–55)
**Height (cm)**	162.5 (160–167)	160 (159–165)
**Body Mass Index (kg/m^2^)**	19.49 (17.55–21.44)	19.51 (18.22–19.51)
**Free Fat Mass (kg)**	45 (41–47)	43 (40–46)
**FEV** **1 ^a^**		
<30% (No., %)	13/23 (57)	2/6 (33)
30–50% (No., %)	6/23 (26)	2/6 (33)
50–70% (No., %)	3/23 (13)	1/6 (17)
>70% (No., %)	1/23 (4)	1/6 (17)
**Anthonisen score ^b^**		
Major (No., %)	12 (24)	18 (41)
Medium (No., %)	27 (54)	8 (18)
Minor (No., %)	11 (22)	18 (41)
**Respiratory distress (No., %)**	17 (34)	29 (66)
**Invasive** **ventilation (No., %)**	7 (14)	13 (30)
**CLCR_CG_ (mL/min)**	62.9 (49.0–76.8)	76.6 (57.5–96.6)
**Concomitant** **medication**		
Diuretic (No., %)	9 (18)	8 (18)
Systemic corticosteroids (No., %)	27 (54)	32 (73)
Inhaled corticosteroids (No., %)	47 (94)	41 (93)
Systemic SABA (No., %)	16 (32)	16 (36)
Inhaled SABA (No., %)	50 (100)	41 (93)
SAMA (No., %)	43 (86)	41 (93)
**Antibiotic regimen**		
0.5 g q12h (No., %)	-	1 (2)
0.5 g q8h (No., %)	-	4 (9)
0.5 g q6h (No., %)	-	7 (16)
1 g q12h (No., %)	1 (2)	8 (18)
1 g q8h (No., %)	39 (78)	24 (55)
1 g q6h (No., %)	1 (2)	-
2 g q12h (No., %)	3 (6)	-
2 g q8h (No., %)	6 (12)	-
**Duration of antibiotherapy—(No., days)**	10 [8–13]	10 [7–14]

^a^ n FEV_1_ (Forced Expiratory Volume in one second): the result is available for 23 patients from the ceftazidime cohort and 6 patients from the imipenem cohort; ^b^ Value measured after 1 day of ceftazidime and imipenem therapy; CLCR_CG_: clearance creatinine estimated by Cockcroft and Gault; SABA: Short-acting bronchodilators; SAMA: Short-acting muscarinic receptor antagonists.

**Table 2:** Population pharmacokinetic model estimates and bootstrap results for ceftazidime and imipenem after intravenous infusion.

Parameters	Final Model				Bootstrap Results		
	Estimates	RSE (%)	*p*		Median	95% Confidence Interval	
						2.5%	97.5%
CEFTAZIDIME							
Vd (L)	23.7	2.96			23.98	22.361	25.66
CL (L/h)	8.74	3.18			8.76	8.185	9.358
β_CLCRCG on CL_	0.485	17.2	2.8 × 10^−8^		0.492	0.328	0.643
ωV (%)	13	32			9.55	3.9	20.1
ωCL (%)	20.8	12.2			19.9	14.8	24.8
b (%)	12.1	16			12.65	7.1	16.6
IMIPENEM							
Vd (L)	15.1	6.07			15.17	13.318	17284
CL (L/h)	7.88	5.35			7.91	7.117	8.771
β_CLCRCG on CL_	0.532	27.2	8.1 × 10^−5^		0.54	0.163	0.949
ωV (%)	10.7	76.4			14.54	6.7	29.2
ωCL (%)	29.4	12.6			28.75	18.9	38.1
b (%)	23.3	12.3			21.3	15.6	26.8

*Vd*, Volume of distribution; CL, Clearance; ωV, Inter-individual variation in the volume of distribution; ωCL, Inter-individual variation in the clearance; *b*, Residual variability; CLCR_CG_, creatinine clearance estimated according to Cockcroft and Gault; β_CL__CR on CL_, the regression coefficient of clearance estimated according to CLCR_CG_ in log scale.

## Data Availability

Not applicable.

## Supplementary Materials

The following are available online at https://www.mdpi.com/article/10.3390/pharmaceutics13040456/s1, Table S1. Selection steps for basic population pharmacokinetic models of ceftazidime and imipenem in our cohorts of patients with acute exacerbations of chronic obstructive pulmonary disease, Table S2. Selection steps for covariates models of ceftazidime and imipenem in our cohorts of patients with acute exacerbations of chronic obstructive pulmonary disease, Table S3. Probability of target attainment (PTA) of ceftazidime administrated as a short-term, extended, and continuous infusion, Table S4. Probability of target attainment (PTA) of imipenem administrated as a short-term, extended, and continuous infusion, Figure S1. Spaghetti plot illustrating ceftazidime (a) and imipenem (b) concentrations versus time, Figure S2. Goodness-of-fit plots of the final model with covariates for ceftazidime and imipenem, Figure S3. Visual Predictive Check plot versus time, Figure S4. Proposed dosing algorithm based on simulation result of ceftazidime and imipenem treatments in Acute Exacerbation of Chronic Obstructive Pulmonary Disease (AECOPD) to obtain 100% *f*T>MIC.
