# Supplementary material for: Population Pharmacokinetics and Dose Optimization of Ceftazidime and Imipenem in Patients with Acute Exacerbations of Chronic Obstructive Pulmonary Disease
Source: Pharmaceutics. 2021 Mar 27;13(4):456. doi: 10.3390/pharmaceutics13040456 (PMC8066993; doi:10.3390/pharmaceutics13040456)
Supplement: Supplementary file 1 [file pharmaceutics-13-00456-s001.pdf]

pharmaceutics

Supplementary Materials: Population Pharmacokinetics and Dose Optimization of Ceftazidime and Imipenem in Patients with Acute Exacerbations of Chronic Obstructive Pulmonary Disease

Thu-Minh Nguyen, Thu-Hue Ngo, Anh-Quan Truong, Dinh-Hoa Vu, Dinh-Chi Le, Ngan-Binh Vu, Tuyet-Nga Can, Hoang-Anh Nguyen, Thu-Phuong Phan, Françoise Van Bambeke, Céline Vidaillac and Quy-Chau Ngo

Table S1. Selection steps for basic population pharmacokinetic models of ceftazidime and imipenem in our cohorts of patients with acute exacerbations of chronic obstructive pulmonary disease.

Project name	Compartment	Error model	BIC
**Ceftazidime**			
PK_C_01	One	constant	620.83
**PK_C_02**	**One**	**proportional**	**613.47**
PK_C_03	One	combine 1	617.63
PK_C_04	One	combine 2	617.14
PK_C_05	Two	constant	632.10
PK_C_06	Two	proportional	625.41
PK_C_07	Two	combine 1	627.87
PK_C_08	Two	combine 2	628.46
**Imipenem**			
PK_I_01	One	constant	566.55
**PK_I_02**	**One**	**proportional**	**552.06**
PK_I_03	One	combine 1	556.34
PK_I_04	One	combine 2	556.38
PK_I_05	Two	constant	577.08
PK_I_06	Two	proportional	567.09
PK_I_07	Two	combine 1	571.34
PK_I_08	Two	combine 2	568.53

BIC: Bayesian information criteria; Based on the BIC values, PK_C_02 or PK_I_02 (**bold**) were selected for the rest of the study.

Table S2. Selection steps for covariates models of ceftazidime and imipenem in our cohorts of patients with acute exacerbations of chronic obstructive pulmonary disease.

Project name	Covariate effect on correlation	OFV reduction	Keep the effect
**CEFTAZIDIME**			
PK_C_09	PK_C_02 + ANTHOSINEN on V	0.18	No
PK_C_10	PK_C_02 + DIURETICS on V	1.4	No
PK_C_11	PK_C_02 + SEX on V	2.78	No
PK_C_12	PK_C_02 + ARDS on V	0.32	No
PK_C_13	PK_C_02 + VENTILATOR on V	0.53	No
PK_C_14	PK_C_02 + AGE on V	0.02	No
PK_C_15	PK_C_02 + CLCR on V	-0.2	No
PK_C_16	PK_C_02 + CREATININ on V	0.21	No
PK_C_17	PK_C_02 + FFM on V	0.68	No
PK_C_18	PK_C_02 + MDRD on V	0.24	No
PK_C_19	PK_C_02 + WEIGHT on V	0.22	No
PK_C_20	PK_C_02 + ANTHOSINEN on CL	0.51	No
PK_C_21	PK_C_02 + DIURETICS on CL	1.28	No
PK_C_22	PK_C_02 + SEX on CL	2.19	No
PK_C_23	PK_C_02 + ARDS on CL	1.52	No
PK_C_24	PK_C_02 + VENTILATOR on CL	1.37	No
PK_C_25	PK_C_02 + AGE on CL	8.2	Yes
**PK_C_26**	**PK_C_02 + CLCR on CL**	**26.26**	**Yes**
PK_C_27	PK_C_02 + CREATININ on CL	12.98	Yes
PK_C_28	PK_C_02 + FFM on CL	4.86	No
PK_C_29	PK_C_02 + MDRD on CL	14.27	Yes
PK_C_30	PK_C_02 + WEIGHT on CL	1.77	No
PK_C_31	PK_C_26 + MDRD on CL	0.21	No
PK_C_32	PK_C_26 + CREATININ on CL	-1.16	No
PK_C_33	PK_C_26 + AGE on CL	0.31	No
**IMIPENEM**			
PK_I_09	PK_I_02 + ANTHOSINEN on V	1.06	No
PK_I_10	PK_I_02 + DIURETICS on V	1.42	No
PK_I_11	PK_I_02 + SEX on V	0.71	No
PK_I_12	PK_I_02 + ARDS on V	4.12	No
PK_I_13	PK_I_02 + VENTILATOR on V	2.7	No
PK_I_14	PK_I_02 + AGE on V	-0.12	No
PK_I_15	PK_I_02 + CLCR on V	-0.54	No
PK_I_16	PK_I_02 + CREATININ on V	-0.47	No
PK_I_17	PK_I_02 + FFM on V	5.5	No
PK_I_18	PK_I_02 + MDRD on V	0.51	No
PK_I_19	PK_I_02 + WEIGHT on V	4.11	No
PK_I_20	PK_I_02 + ANTHOSINEN on CL	7.28	Yes

**Project name**	**Covariate effect on correlation**	**OFV reduction**	**Keep the effect**
PK_I_21	PK_I_02 + DIURETICS on CL	3.73	No
PK_I_22	PK_I_02 + SEX on CL	-0.09	No
PK_I_23	PK_I_02 + ARDS on CL	0.49	No
PK_I_24	PK_I_02 + VENTILATOR on CL	-0.11	No
PK_I_25	PK_I_02 + AGE on CL	8.23	Yes
**PK_I_26**	**PK_I_02 + CLCR on CL**	**11.89**	**Yes**
PK_I_27	PK_I_02 + CREATININ on CL	6.37	No
PK_I_28	PK_I_02 + FFM on CL	-0.38	No
PK_I_29	PK_I_02 + MDRD on CL	7.84	Yes
PK_I_30	PK_I_02 + WEIGHT on CL	-0.1	No
PK_I_31	PK_I_26 + AGE on CL	3.35	No
PK_I_32	PK_I_26 + MDRD on CL	0,02	No
PK_I_33	PK_I_26 + ANTHIOSINEN on CL	3.35	No

PK_C_26 and PK_I_26 (**bold**) were selected because showing highest OFV reduction (minimum OFV).

Table S3. Probability of target attainment (PTA) of ceftazidime administrated as short-term, extended and continuous infusion.

**Ceftazidime**		**100% *f*T>MIC Probability of target attainment**									**60% *f*T>MIC Probability of target attainment**								
		**MIC (mg/L)** [a]									**MIC (mg/L)** [a]								
**$CLCR_{CG}$**	**Dose regimen**	**0.125**	**0.25**	**0.5**	**1**	**2**	**4**	**8**	**16**	**32**	**0.125**	**0.25**	**0.5**	**1**	**2**	**4**	**8**	**16**	**32**
30-60 mL/min	1g q12h (SI)	94.6	87.2	64.9	40.3	14.2	1.4	0.1	0	0	100	99.9	99.5	97.6	85.7	44.3	5.3	0.1	0
	2g q12h (SI)	98.1	95.2	87.7	71.2	42.8	13.9	1.2	0	0	100	100	100	99.6	97.6	85.8	47	4.3	0
	1g q8h (SI)	100	99.7	98.9	92.7	72.1	34.8	5.6	0	0	100	100	100	100	99.7	95.4	58.8	3.8	0
	2g q8h (SI)	100	99.9	99.6	98.3	94	75.3	34.2	4	0	100	100	100	100	100	99.7	96.2	58.7	3.1
	1g q12h (EI)	98.7	95.3	85.7	62.7	27.7	4	0	0	0	100	100	100	99.9	98.2	76.4	13.5	0	0
	2g q12h (EI)	99.8	99	95.6	83.9	60.3	27.4	3.1	0.1	0	100	100	100	100	100	97.9	74.1	13.6	0
	1g q8h (EI)	100	100	100	99	92.2	65.4	15.4	0.1	0	100	100	100	100	100	99.9	86.5	8.9	0
	2g q8h (EI)	99.9	99.9	99.9	99.8	99.3	94.5	67.4	16.1	0.3	100	100	100	100	100	99.9	99.8	87.8	10.1
	6g q24h (CI)	100	100	100	100	100	100	100	99.3	26.4	100	100	100	100	100	100	100	99.3	26.4
60-90 mL/min	1g q12h (SI)	81.6	64.7	38.3	14.2	1.6	0	0	0	0	99.8	99.3	96.7	87.4	61.9	17.2	0.3	0	0
	2g q12h (SI)	91.7	81.1	64	39	15.7	2.5	0	0	0	100	100	99.8	97.7	87.1	61.4	17.6	0.3	0
	1g q8h (SI)	99.8	97.8	91.1	77.8	47.1	12.3	0.4	0	0	100	100	100	100	98	82.8	28.5	0.2	0
	2g q8h (SI)	99.9	99.6	97.4	91.9	76.4	44	10.8	0.5	0	100	100	100	100	99.9	97.4	81.9	27.7	0.5
	1g q12h (EI)	92.8	80.2	59.4	32	7.5	0.6	0	0	0	100	100	99.7	98.7	87.7	44.6	2.1	0	0
	2g q12h (EI)	96.9	92	81.1	60.1	31.8	8.2	0.3	0	0	100	100	100	99.9	98.5	87.6	45.6	2.6	0
	1g q8h (EI)	100	99.9	99.3	95.8	77.5	34.1	2.7	0	0	100	100	100	100	100	99.1	64.1	0.9	0
	2g q8h (EI)	100	100	99.9	99.2	94.6	75.9	34.6	2.5	0.1	100	100	100	100	100	100	98.8	61.1	1.4
	6g q24h (CI)	100	100	100	100	100	100	100	96.3	6.7	100	100	100	100	100	100	100	96.3	6.7
>90 mL/min	1g q12h (SI)	49	30.4	13.2	3	0.2	0	0	0	0	97.5	92.8	82.4	58	26.7	3.6	0	0	0
	2g q12h (SI)	67.4	50.1	30.4	15.4	3.6	0.3	0	0	0	99.4	98.3	95.4	84.7	60.9	27.1	4.1	0.2	0
	1g q8h (SI)	94.1	86.2	71.2	46.2	18	1.9	0	0	0	100	99.6	99.1	97	85.7	51.7	7	0	0
	2g q8h (SI)	97.8	94.9	86.8	70.1	44.7	14.6	1.5	0	0	100	100	99.9	99.4	97.3	86.2	50	6.3	0
	1g q12h (EI)	69	49.4	27.6	8.6	1.7	0	0	0	0	99.9	99.6	97.2	88.5	59.1	15.1	0.2	0	0
	2g q12h (EI)	83.2	68.9	50.5	27	8.6	1.6	0	0	0	100	99.9	99.5	96.5	87.2	58	15.5	0.3	0
	1g q8h (EI)	99.8	97.9	91.4	74.6	44.9	10.5	0.4	0	0	100	100	100	100	99.9	89.1	30.1	0.1	0
	2g q8h (EI)	100	99.7	98.3	91.6	76.1	44.1	10.6	0.4	0	100	100	100	100	100	99.8	90	26.9	0.2
	6g q24h (CI)	100	100	100	100	100	100	100	77.4	1	100	100	100	100	100	100	100	77.4	1

[a] According to the Clinical and Laboratory Standards Institute 2020 (CLSI): S, MIC ≤ 8 mg/L (black); I, MIC = 16 mg/L (orange); R, MIC ≥ 32 mg/L (red). For 6g q24h, renew infusion solution thrice daily with following initial loading dose of 2 g bolus. PTA ≥ 90% was highlighted in green.

Table S4. Probability of target attainment (PTA) of imipenem administrated as short-term, extended and continuous infusion.

Imipenem		100% *f*T>MIC Probability of target attainment MIC (mg/L) [a]									40% *f*T>MIC Probability of target attainment MIC (mg/L) [a]								
**CLCRcg**	**Dose regiment**	**0.125**	**0.25**	**0.5**	**1**	**2**	**4**	**8**	**16**	**32**	**0.125**	**0.25**	**0.5**	**1**	**2**	**4**	**8**	**16**	**32**
30-60 mL/min	0.75g q8h (SI)	96.9	93.3	84.5	69.2	44.3	15.8	2.3	0.2	0	100	100	100	100	99.4	98	80.6	17.6	0.2
	0.5g q6h (SI)	99.4	98.6	96.1	85.9	62.5	30.1	5.6	0	0	100	100	100	100	100	99.1	80.5	14	0
	1g q8h (SI)	97.9	95	89.1	77.5	54.3	25.8	8.5	0.8	0.1	100	100	100	100	100	99.5	92.5	45.7	2.3
	0.75g q6h (SI)	99.8	99.6	97.8	92.1	77.6	52.4	17.9	1	0	100	100	100	100	100	100	97.3	55	1.6
	1g q6h (SI)	99.9	99.7	98.7	96.2	86.9	62.8	31.5	4.5	0.3	100	100	100	100	100	99.9	99.3	82.1	10.6
	0.75g q8h (EI)	99.7	98.7	95	87.2	68.1	35.4	9	0.4	0	100	100	100	100	100	100	96.7	29.7	0
	0.5g q6h (EI)	100	99.7	99.5	97.6	89.3	60.9	15.9	0.4	0	100	100	100	100	100	100	93.1	12.3	0
	1g q8h (EI)	99.4	99	97.6	91.7	77.7	50	18.1	2.2	0	100	100	100	100	100	100	99.7	67	2.4
	0.75g q6h (EI)	100	99.9	99.8	99.1	96.5	81.1	44.1	7.3	0	100	100	100	100	100	100	100	67	2.6
	1g q6h (EI)	100	100	99.9	99.7	98.2	92.5	64.8	19.7	0.6	100	100	100	100	100	100	100	95.6	17
	4g q24h (CI)	100	100	100	100	100	100	100	81.6	8.2	100	100	100	100	100	100	100	81.6	8.2
60-90 mL/min	0.75g q8h (SI)	86.9	75.8	58.8	38.7	16.4	4	0.3	0	0	100	100	100	99.9	97.8	89	51.5	4.3	0
	0.5g q6h (SI)	96.4	90.5	79.1	60.8	32.6	9.3	0.4	0	0	100	100	100	100	99.7	93.7	52.6	2.1	0
	1g q8h (SI)	87.9	78.3	65	47.8	25.9	8.9	1.3	0.1	0	100	100	100	99.9	98.9	92.6	70	17.8	0.3
	0.75g q6h (SI)	98.8	95.8	88.4	76.2	51.2	24.5	4.6	0.2	0	100	100	100	100	99.9	99.4	84.8	27.1	0.3
	1g q6h (SI)	98.6	97.2	92.7	82.1	60.6	34.1	10.4	0.8	0.1	100	100	100	100	99.9	99.5	95.3	53.4	3.8
	0.75g q8h (EI)	96.8	91.9	82.6	65.9	39.4	14.1	1.3	0.1	0	100	100	100	100	100	99.9	86.3	9.1	0.1
	0.5g q6h (EI)	99.9	99.4	97.5	89.3	67.5	30.7	4.4	0	0	100	100	100	100	100	100	76.7	3.5	0
	1g q8h (EI)	98.8	95.1	86.6	71	46.2	20	3.2	0.1	0	100	100	100	100	100	100	98.7	33.8	0.1
	0.75g q6h (EI)	99.8	99.8	99	95.8	84.8	53.8	16.3	0.5	0	100	100	100	100	100	100	99.2	35.6	0.1
	1g q6h (EI)	100	99.9	99.5	97.8	89.6	66.5	29.4	4.4	0	100	100	100	100	100	100	100	75.9	2.3
	4g q24h (CI)	100	100	100	100	100	100	100	54.9	1.3	100	100	100	100	100	100	100	54.9	1.3
>90 mL/min	0.75g q8h (SI)	82.6	73.3	58.7	40.1	21.4	7.3	0.7	0	0	100	100	99.9	99.4	96.6	85.1	52.1	8	0
	0.5g q6h (SI)	94.5	89.5	79.4	60.9	37	13.2	1.8	0	0	100	100	100	99.9	99.2	92.3	55	4.3	0
	1g q8h (SI)	87.5	79.5	66.9	51.1	29.9	13.6	2.9	0.3	0	100	100	100	99.7	97.9	93	71.9	25	0.9
	0.75g q6h (SI)	97.2	93.1	85.5	71.7	52.3	26.6	6.5	0.8	0	100	100	100	99.9	99.7	98	82.6	29.3	0.8
	1g q6h (SI)	98	95.9	90.7	79.4	62.4	36.4	12.2	1.5	0	100	100	100	100	100	99.4	93.1	56.1	4.1
	0.75g q8h (EI)	94.8	89.3	80.3	63.5	40.7	15.1	2.2	0.1	0	100	100	100	100	100	99.7	82.5	10.8	0.1
	0.5g q6h (EI)	99.7	98.7	95.1	86	64.6	33.5	6.1	0.3	0	100	100	100	100	100	100	72.4	4.9	0
	1g q8h (EI)	96.8	92.9	83.2	69.8	50.2	24.6	7.1	0.6	0	100	100	100	100	100	100	97.2	39.4	0.7
	0.75g q6h (EI)	99.9	98.9	97.4	93.1	80.2	55.8	21.4	2.7	0	100	100	100	100	100	100	98.4	39.2	0.8
	1g q6h (EI)	99.9	99.7	98.9	96.2	86.6	65.2	31.3	7.2	0.4	100	100	100	100	100	100	99.9	74.6	5.4
	4g q24h (CI)	100	100	100	100	100	100	96.2	32.5	0.5	100	100	100	100	100	100	96.2	32.5	0.5

[a] According to the Clinical and Laboratory Standards Institute 2020 (CLSI): S, MIC ≤ 2 mg/L (black); I, MIC = 4 mg/L (orange); R, MIC ≥ 8 mg/L (red). For 4g q24h, renew infusion solution six times a day to ensure stability of imipenem. PTA ≥ 90% was highlighted in green.

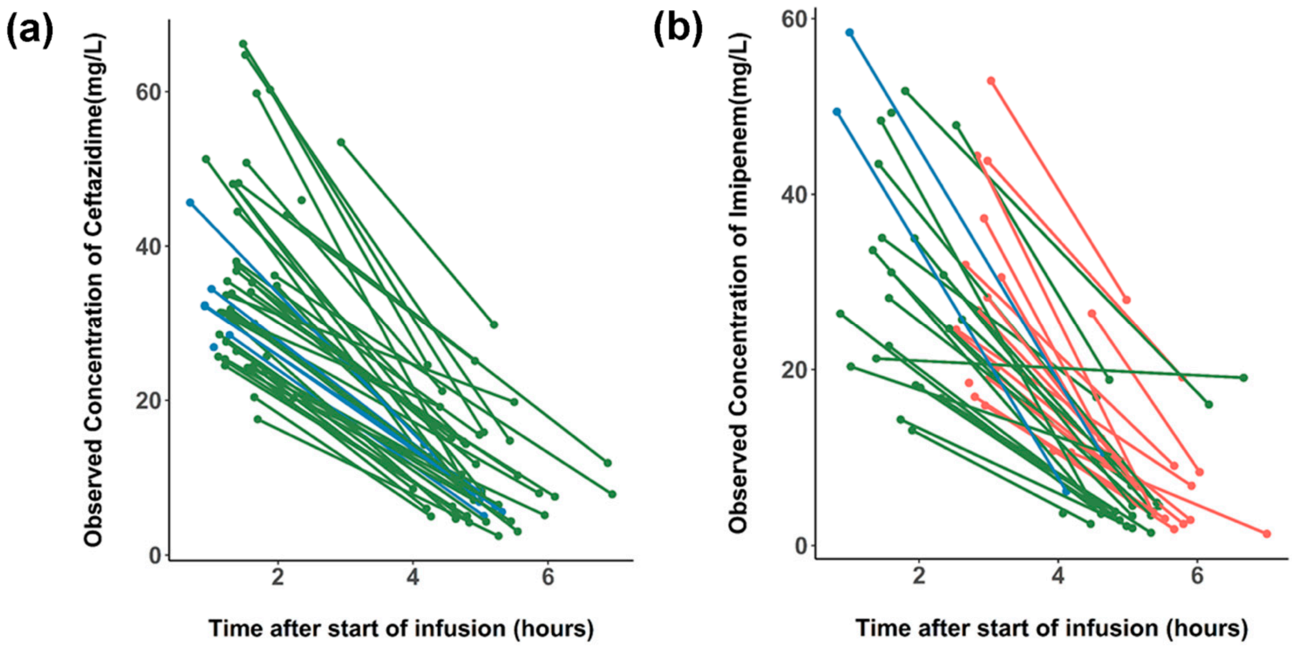

Figure S1. Spaghetti plot illustrating ceftazidime (**a**) and imipenem (**b**) concentrations versus time. Each line represents drug concentration (mg/L) measured in one participant at least 30 minutes post-dose and one or two hours before next-dose. Patients received difference infusion time: <30 mins (blue), 30 mins. to 120 mins (green) and >120 mins (red). Due to the withdrawal of patients after first sample (5), patient's transfer (1) or patient's emergency care (1), seven patients had only one measured concentration and appear as a single dot on the plots.

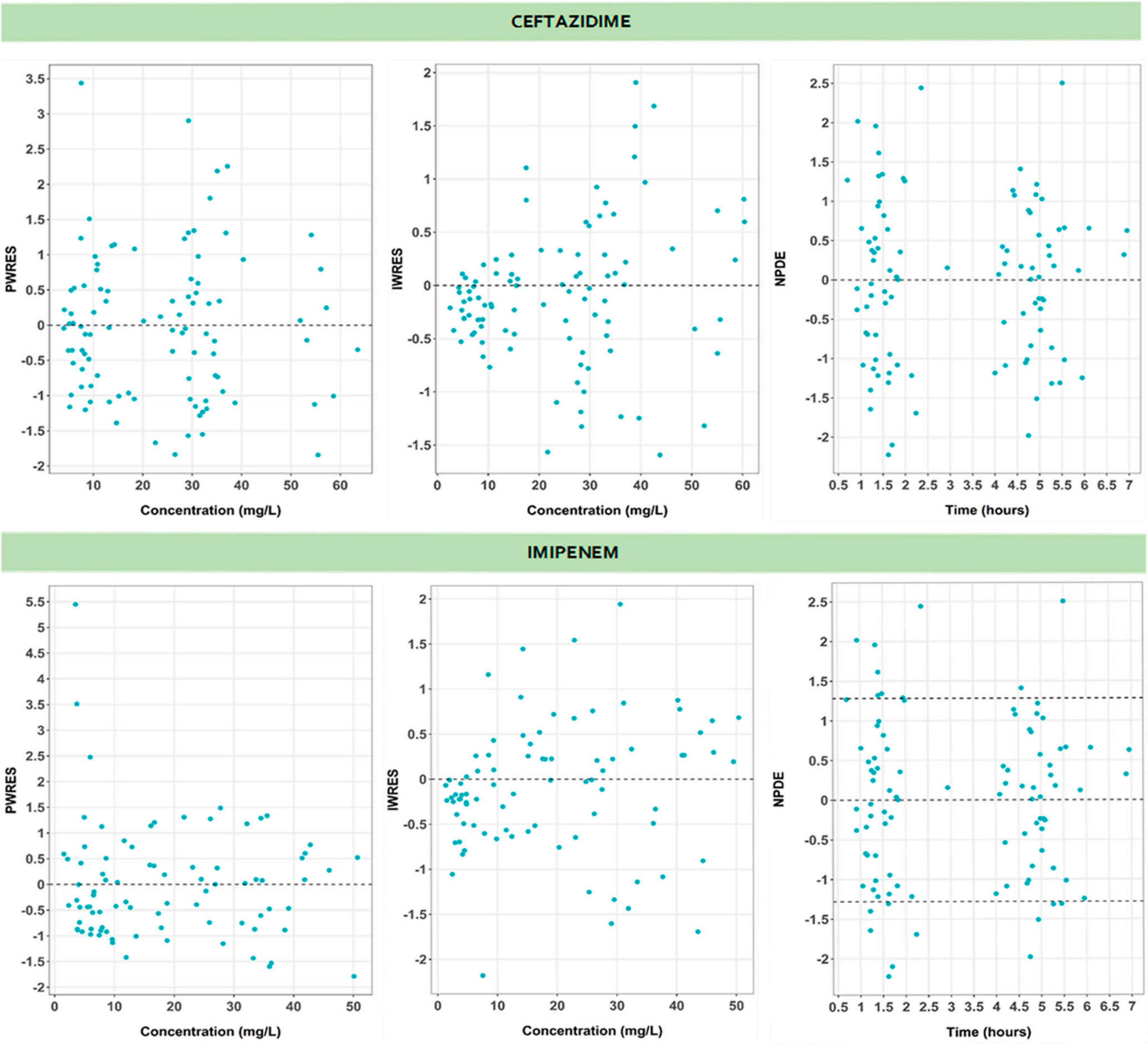

Figure S2. Goodness-of-fit plots of the final model with covariates for ceftazidime and imipenem. Left panels show the population weighted residuals (PWRES) plotted versus the population predicted concentrations (mg/L). The middle panels show Table 3. Proposed dosing algorithm based on simulation result of ceftazidime and imipenem treatments in Acute Exacerbation of Chronic Obstructive Pulmonary Disease (AECOPD) with the aim to obtain 100% *f*T>MIC. PA: *Pseudomonas aeruginosa*; (S): Susceptible ; (I): Intermediate resistance; IMI: imipenem; CAZ: ceftazidime; $CLCR_{CG}$: Clearance creatinine according to Cockcroft and Gault equation; dose follow 3-hours extended infusion with dose interval of 6 hours (q6h), 8 hours (q8h), 12 hours (q12h); CI: continuous infusion.

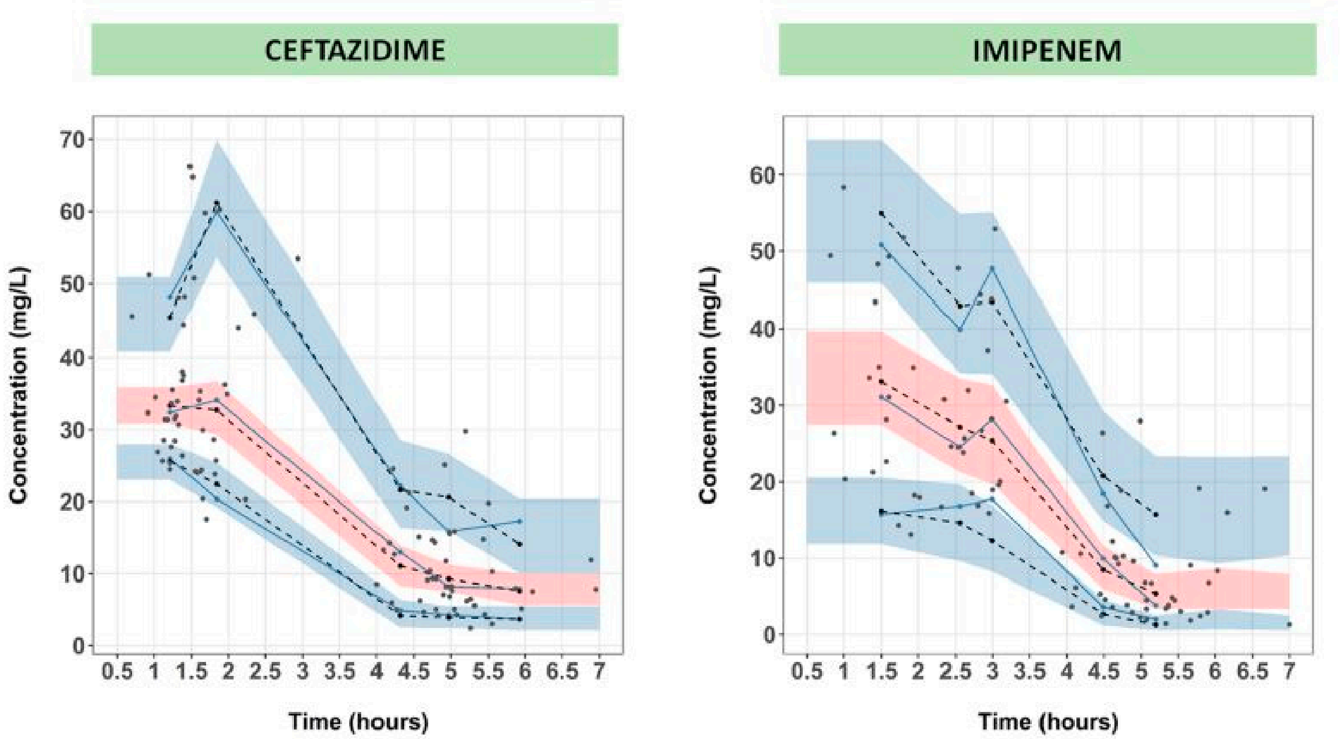

Figure S3. Visual Predictive Check plot versus time. The grey solid lines indicate the 10th, 50th, and 90th percentiles of the observed data. The grey black dashed lines indicate the 10th, 50th, and 90th percentiles of simulated data. The shaded grey and pink areas represent 90% prediction intervals from the corresponding percentiles as predicted by the model.

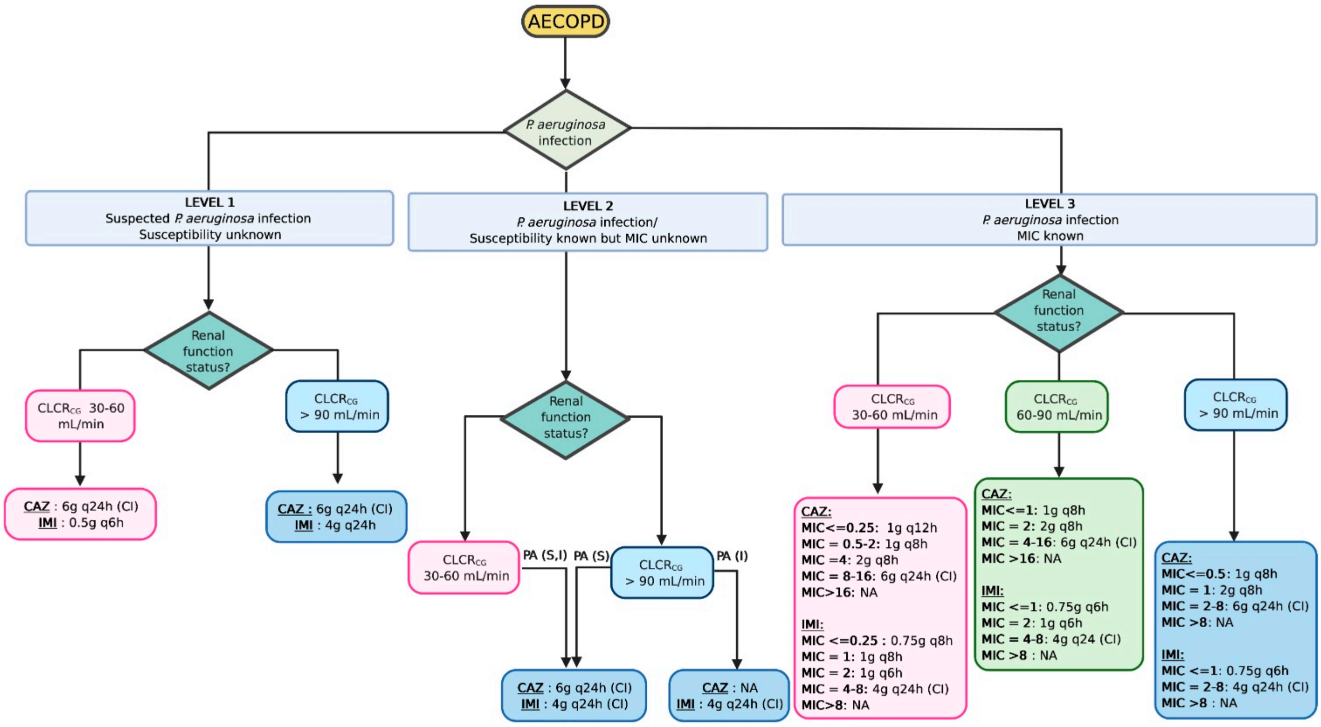

Figure S4. Proposed dosing algorithm based on simulation result of ceftazidime and imipenem treatments in Acute Exacerbation of Chronic Obstructive Pulmonary Disease (AECOPD) with the aim to obtain 100% *f*T>MIC. PA: *Pseudomonas aeruginosa*; (S): Susceptible ; (I): Intermediate resistance; IMI: imipenem; CAZ: ceftazidime; $CLCR_{CG}$: Clearance creatinine according to Cockcroft and Gault equation; dose follow 3-hours extended infusion with dose interval of 6 hours (q6h), 8 hours (q8h), 12 hours (q12h); CI: continuous infusion.
